# The Role of Vitamin E in the Treatment of NAFLD

**DOI:** 10.3390/diseases6040086

**Published:** 2018-09-24

**Authors:** Brandon J. Perumpail, Andrew A. Li, Nimy John, Sandy Sallam, Neha D. Shah, Waiyee Kwong, George Cholankeril, Donghee Kim, Aijaz Ahmed

**Affiliations:** 1 Drexel University College of Medicine, Philadelphia, PA 19129, USA; brandonperumpail@gmail.com; 2 Department of Medicine, Stanford University School of Medicine, Stanford, CA 94305, USA; andrewli@stanford.edu; 3 Division of Gastroenterology and Hepatology, Stanford University School of Medicine, Stanford, CA 94305, USA; nionnj@gmail.com (N.J.); SSallam@stanfordhealthcare.org (S.S.); NeShah@stanfordhealthcare.org (N.D.S.); WKwong@stanfordhealthcare.org (W.K.); georgetc@stanford.edu (G.C.); dhkimmd@stanford.edu (D.K.)

**Keywords:** NAFLD, NASH, nonalcoholic fatty liver disease, vitamin E alpha-tocopherol

## Abstract

There has been a growing interest in the role of vitamin E supplementation in the treatment and/or prevention of nonalcoholic fatty liver (NAFLD). We performed a systematic review of the medical literature from inception through 15 June 2018 by utilizing PubMed and searching for key terms such as NAFLD, vitamin E, alpha-tocopherol, and nonalcoholic steatohepatitis (NASH). Data from studies and medical literature focusing on the role of vitamin E therapy in patients with NAFLD and nonalcoholic steatohepatitis (NASH) were reviewed. Most studies assessing the impact of vitamin E in NAFLD were designed to evaluate patients with NASH with documented biochemical and histological abnormalities. These studies demonstrated improvement in biochemical profiles, with a decline in or normalization of liver enzymes. Furthermore, histological assessment showed favorable outcomes in lobular inflammation and hepatic steatosis following treatment with vitamin E. Current guidelines regarding the use of vitamin E in the setting of NAFLD recommend that vitamin E-based treatment be restricted to biopsy-proven nondiabetic patients with NASH only. However, some concerns have been raised regarding the use of vitamin E in patients with NASH due to its adverse effects profile and lack of significant improvement in hepatic fibrosis. In conclusion, the antioxidant, anti-inflammatory, and anti-apoptotic properties of vitamin E accompanied by ease-of-use and exceptional tolerability have made vitamin E a pragmatic therapeutic choice in non-diabetic patients with histologic evidence of NASH. Future clinical trials with study design to assess vitamin E in combination with other anti-fibrotic agents may yield an additive or synergistic therapeutic effect.

## 1. Introduction

Nonalcoholic fatty liver disease (NAFLD) and its progressive subset, nonalcoholic steatohepatitis (NASH) affect a significant proportion of the population. However, no medications are currently approved for its treatment; the primary therapeutic intervention is lifestyle modification through diet and exercise [1,2,3]. The focus of this review article is to discuss the role of vitamin E in patients with NASH, a subset of NAFLD characterized by ongoing hepatic inflammation and fibrosis which can progress to cirrhosis and end-stage liver disease necessitating liver transplantation. 

Vitamins with antioxidant properties have been known to provide health benefits [4]. They have the ability to act through various mechanisms to decrease the levels of reactive oxygen species in the body and prevent oxidative damage in the cell that can lead to cellular senescence and apoptosis [4]. These properties may retard the progression of liver injury and may even facilitate the reversal of hepatic fibrosis in patients with NAFLD who are at risk for developing NASH, as oxidative stress has been implicated as one of the key pathways in NASH pathogenesis [3]. In the setting of metabolic syndrome, the increased delivery of fatty acids to the liver results in amplified oxidative stress through fatty acid oxidation and oxidative phosphorylation [3]. This produces an environment high in reactive oxygen species that can cause hepatocyte injury and progressive hepatic damage [3]. Thus, the supplementation with antioxidant vitamins provides a potential therapeutic option for patients with NAFLD [2]. Vitamin E, a key fat-soluble antioxidant, has the most evidence for its ability to provide a therapeutic benefit [2]. The current guidelines by the American Association for the Study of Liver Disease (AASLD) and the European Association for the Study of the Liver (EASL) have recommended the use of vitamin E in patients with biopsy-proven NASH and without diabetes. However, current data on the efficacy and safety of vitamin E has not been considered sufficient to expand this recommendation to diabetic patients with NASH [1,2]. Vitamin E has the ability to reduce the oxidative stress, retard the pathogenesis of NASH, and therefore, provide a viable therapeutic option in the treatment of patients with NASH [3].

## 2. Mechanism of Action and Therapeutic Role of Vitamin E in Patients with NASH

### 2.1. Antioxidant Effects of Vitamin E

Oxidative stress is created when the production of reactive oxygen species (ROS) overwhelms antioxidative pathways [5,6]. Vitamin E is one of the most powerful chain-breaking antioxidants in the human body [6,7]. There are many forms including alpha, beta, and gamma tocopherols and tocotrienols; however, the alpha-tocopherol form is the most prevalent and active in tissue and human plasma [6,7]. It has inherent antioxidative activity as it can donate a hydrogen ion from its chromanol ring to scavenge lipid peroxyl radicals [6,7,8]. This radical scavenging ability of vitamin E is not limited to ROS, as it is also active against reactive nitrogen species (RNS), which has been a separate area of recent interest [9]. Furthermore, vitamin E interacts with other cellular components and may help foster the antioxidative environment [6,8]. Superoxide dismutase (SOD), a crucial antioxidative enzyme in the body, can partition superoxide radicals into oxygen or hydrogen peroxide and is increased with vitamin E supplementation [6,10,11,12,13]. Vitamin E also increases the actions of other antioxidative enzymes such as catalase and glutathione peroxidase [6,11,12,13]. Through gene expression modulation, dietary vitamin E can decrease c-myc and transform growth factor-alpha expression, leading to decreased nitric oxide synthase (iNOS) and the reduced form of nicotinamide adenine dinucleotide phosphate (NADPH) oxidase which are major contributors to oxidative stress [13,14]. In addition, vitamin E has the ability to repress peroxidation and inhibit the expression of transforming growth factor-beta which has been associated with hepatic fibrosis and hepatocyte apoptosis by activating of hepatic stellate cells [15]. 

### 2.2. Anti-Inflammatory and Anti-Apoptotic Properties of Vitamin E

**Figure 1:**
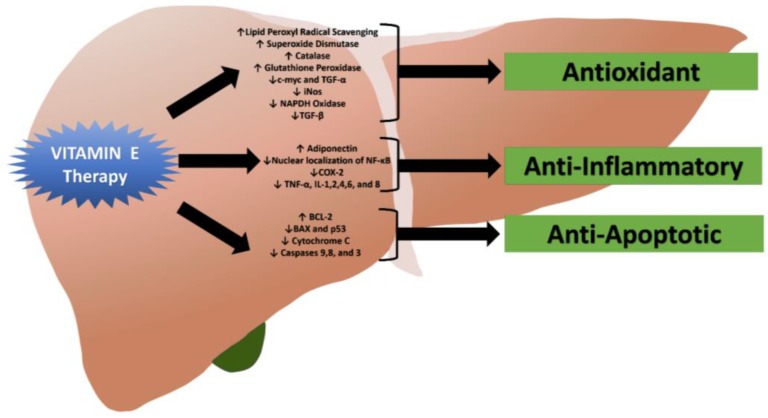
The effects of vitamin E in patients with nonalcoholic steatohepatitis (NASH).

In addition to its antioxidative capabilities, vitamin E has other therapeutic effects that can retard hepatic fibrosis and may prevent cirrhosis by modulating inflammatory response, cell injury, cellular signaling, and cellular proliferation [3,16]. Studies have linked vitamin E supplementation with increased adiponectin mRNA and protein levels [17,18]. Adiponectin is an important molecule and works by suppressing hepatic fatty acid synthesis and reducing inflammation in patients with NASH [17]. Vitamin E is also a powerful player in cell death and apoptosis pathways [17,19]. Recent research has found that it reduced the rates of apoptosis by alleviating the intracellular mitochondrial membrane potential, increasing levels of anti-apoptotic protein BCL-2, and decreasing levels of pro-apoptotic proteins BAX and p53 [19]. Furthermore, it was able to decrease the activity of caspase-9 and cytochrome C in the mitochondrial apoptosis pathway, as well as caspase-8 and caspase-3 in the Fas/FasL apoptotic pathway [19]. By preventing the nuclear localization of NF-κB, decreasing COX-2 expression, and suppressing the expression of cytokines TNF-alpha, IL-1, IL-2, IL-4, IL-6, and IL-8, vitamin E is able to dampen the inflammatory response in NAFLD [8,13,20]. Figure 1 illustrates the therapeutic effects of vitamin E in patients with NASH.

## 3. Clinical Use of Vitamin E in Patients with NASH

Several studies have been conducted to explore the potential role of vitamin E-based therapy in patients with NASH. Various measurements were used to evaluate the effect of vitamin E on biochemical parameters (liver enzyme levels) and hepatic histology (steatosis, lobular inflammation, balloon degeneration, and fibrosis). These clinical studies explored the efficacy of vitamin E monotherapy, as well as dual therapy in conjunction with other potential therapeutic agents in patients with NASH [15,21,22,23,24,25,26,27,28,29,30,31,32,33,34,35,36,37,38,39,40,41,42].

### 3.1. Biochemical Studies

At least seven studies that assessed serum levels of aspartate aminotransferase (AST) and alanine aminotransferase (ALT) to evaluate liver function demonstrated a significant improvement with vitamin E treatment in patients with NASH [21,22,23,24,25,26,27,28]. Kawanaka et al. noted a reduction in ALT levels in response to treatment with vitamin E 300 mg per day orally in all (*n* = 10) patients with NASH who previously failed dietary modifications [22]. These results were reproduced in other clinical trials [24,26]. A Turkish study noted an improvement in serum aminotransferase levels in the vitamin E and C combination therapy group of 28 patients with NASH; the response was more pronounced in the vitamin E and C group versus the other treatment group using ursodeoxycholic acid [24]. A meta-analysis of studies that measured changes in AST and ALT levels with vitamin E therapy in the setting of NAFLD, NASH, and chronic hepatitis C noted improvement in all three conditions, with greatest mean improvement in the NASH group [27]. However, the small number of NAFLD studies and variation in data made the analysis of these patients statistically insignificant [27]. 

### 3.2. Histological Studies

Several clinical trials studied the efficacy of vitamin E in patients with NASH by assessing the histological response including measures of hepatic inflammation (steatosis, ballooning degeneration, and lobular inflammation) and fibrosis before and after treatment [15,29,30,31,32,33,34,35,36,37,38,39,40,41,42]. Two well-known clinical trials are the ‘Pioglitazone, Vitamin E, or Placebo for Nonalcoholic Steatohepatitis’ (PIVENS) trial by Sanyal et al., and the ‘Treatment of NAFLD in Children’ (TONIC) pediatric trial by Lavine et al. [35,37]. An initial pilot study by Sanyal et al. compared the efficacy of vitamin E at 400 IU/day versus a combination therapy of the same dose of vitamin E plus pioglitazone 30 mg/day with each group consisting of ten patients [30]. This study demonstrated improved steatosis in both groups but only noted significant decreases in hepatocyte ballooning and pericellular fibrosis compared to baseline in the combination therapy group [30]. In the subsequent PIVENS trial, Sanyal et al. compared each treatment as monotherapy versus the placebo group in patients with NASH [35]. After 96 weeks, the primary outcome, defined as the rate of improvement in NASH, was assessed against the placebo and the vitamin E group (800 IU/day) showed a statistically significant improvement (34%) while the pioglitazone group (30 mg/day) failed to demonstrate a significant improvement [35]. Treatment with vitamin E also showed a reduction in activity score for NASH with improved ALT, hepatic steatosis, lobular inflammation, and hepatocellular ballooning; however, no significant improvement in fibrosis was noted [35]. Reassessment of the data from the PIVENS trial by Hoofnagle et al. calculated that the ability of vitamin E to reduce ALT and hepatocellular ballooning was independent of the weight loss by the study participants, further strengthening the potential role of vitamin E as a therapeutic agent [39].

Lavine et al. conducted the larger, multicenter TONIC trial for pediatric patients [37]. An earlier pilot study by the same group studied pediatric patients with NASH who were prescribed vitamin E doses ranging from between 400 and 1200 IU daily for 4 to 10 months and were evaluated by measurements in serum aminotransferases [21]. The TONIC clinical trial included three arms comparing vitamin E (800 IU/day), metformin (1000 mg/day), and placebo in 173 patients with biopsy-proven NAFLD/NASH in children and adolescent from 10 centers for 96 weeks [37]. Although all groups achieved the primary outcome of sustained reduction in ALT levels, both vitamin E and metformin failed to do so more superiorly than placebo. However, vitamin E treatment led to the greatest resolution in NASH through improvement in hepatocellular ballooning [37].

A meta-analysis of five randomized controlled trials in patients with NASH who were treated with vitamin E used a random effect analysis to assess if vitamin E had a therapeutic effect [42]. The results of the meta-analysis revealed that vitamin E significantly reduced AST, ALT, steatosis, inflammation, and hepatocellular ballooning [42]. Furthermore, in adults with NASH, vitamin E also reduced hepatic fibrosis [42]. 

### 3.3. Vitamin E Combination Therapies for Patients with NASH

Some studies suggested that vitamin E be used in combination with other therapeutic agents as a treatment approach for patients with NASH [24,28,29,32,36,38,40,41]. The combination of vitamin E with vitamin C as a combined antioxidant therapy for patients with NASH has been pursued [24,29,40,41]. A double-blind study by Harrison et al., utilizing combined vitamin E (1000 IU) and vitamin C (1000 mg) daily regimen for six months, demonstrated an improvement in hepatic fibrosis scores in patients with NASH compared to the placebo group [29]. Kawanaka et al. used lower doses of vitamin E and vitamin C, 300 IU and 300 mg, respectively, for 12 months in patients NASH and noted an improvement in ALT, necroinflammatory activity, and fibrosis compared to pretreatment [40]. The combination therapy of ursodeoxycholic acid (UDCA) and vitamin E was explored by Dufour et al., and more recently by Pietu et al., with both studies noting improved AST, ALT, and NAFLD activity scores due to decreased hepatic steatosis [32,38]. Foster et al. measured the beneficial effects of atorvastatin (20 mg), Vitamin C (1 g), and Vitamin E (1000 IU) daily versus placebo on NAFLD probability based upon computed tomography scanned liver to spleen ratio and calculated the treatment group to have decreased probability and improved ratios [36]. In the most recent vitamin E randomized control trial in patients with NASH, Zohrer et al. observed improved ALT and hepatic steatosis in children treated with docosahexaenoic acid, choline, and vitamin E together in combination [28]. These studies propose that vitamin E therapy can be used in conjunction with other treatment targets to improve the response rate by a synergistic or additive therapeutic effect. All the major studies are summarized in Table 1. 

### 3.4. Optimal Strategy for Utilizing Vitamin E in Patients with NASH

Current data metrics are promising and support the use of vitamin E in patients with non-diabetic NASH. However, it is important to recognize that vitamin E should not be regarded as the first-line treatment approach [25,26,34]. The beneficial effects of vitamin E in this patient population are comparable to, but not more effective than, lifestyle interventions, such as diet modification and exercise. Therefore, vitamin E therapy should be considered as a treatment option if lifestyle modifications fail to produce the expected results due to noncompliance or ineffectiveness [25,26,34]. Nobili et al. compared the effect of improved nutrition and physical activity on ALT levels in pediatric patients diagnosed with NAFLD with and without a combination of vitamin E (600 IU/day) and vitamin C (500 mg/day) supplementation, demonstrating that the addition of vitamins E and C did not improve liver function tests more than lifestyle intervention alone [25]. In a follow-up study, Nobili et al. studied the hepatic histology and established the same conclusion [34]. A Chinese study noted significant improvement in liver function tests in 19 obese children with NAFLD treated with 100 mg of vitamin E daily for a month, nine of whom experienced a normalization of AST and ALT levels. However, the comparative treatment arm with 19 children managed with lifestyle interventions demonstrated relatively superior improvement in liver enzymes with 10 children demonstrating normalization of AST and ALT levels [26]. 

An analysis by Chang et al. used samples from the PIVENS study to identify metabolomic profile changes that can predict the histological response [43]. The study revealed that patients with significant changes in TNF-alpha, gamma-glutamyl leucine, and gamma-glutamyl valine during treatment showed histological improvement [43]. Further studies in this area can help determine which patients would be more likely to experience successful vitamin E treatment [43]. In the PIVENS trial, Sanyal et al. noted that 50% of patients failed to demonstrate a biochemical response to vitamin E, therefore making AST and ALT unreliable predictors of the effectiveness of vitamin E, and, thus, stressing the need for a liver biopsy [35]. The data from Chang et al. can potentially be used to reduce the need for a liver biopsy in assessing the response to vitamin E-based treatment in patients with NASH [43]. 

## 4. Potential Adverse Effects of Vitamin E 

Despite the beneficial effects of vitamin E, various meta-analyses have raised concerns against the long-term use of vitamin E [44,45,46,47]. One analysis noted doses greater than 400 IU/day to be associated with increased all-cause mortality, while another reported vitamin E below 5500 IU/day to have no effect on all-cause mortality. The Cochrane Systematic Review reports that vitamin E can be associated with increased risk of all-cause mortality. However, Oliver et al. debated the validity of the analytical approach in this study and recommended that a more accurate method be used to assess the adverse effects resulting from vitamin E [48,49]. Other concerns associated with vitamin E treatment include a minor risk of prostate cancer and hemorrhagic stroke [50,51].

## 5. Role of Other Vitamins, Minerals, and Emerging Strategies in Patients with NAFLD 

### 5.1. Vitamins C, D, and A

Although vitamin E has been the most studied with NAFLD, other vitamins have gained interest as well. However, direct comparisons between these vitamins and vitamin E have not been done to deem whether these vitamins have a greater or lesser therapeutic effect than vitamin E. Vitamin C is very similar to vitamin E in that it is also a strong antioxidant and thus can decrease the oxidative stress seen in patients with NAFLD and NASH. Vitamin C has been used with vitamin E as a combination antioxidative treatment in several studies to treat NASH and NAFLD [24,29,40,41]. A cross-sectional study noted an inverse relationship between the incidence of NAFLD and dietary vitamin C intake in older adults alluding that dietary supplementation can provide a protective role against NAFLD [52].

Vitamin D has recently gained more attention as researchers have noticed the high correlation of vitamin D deficiency and NAFLD [53,54,55,56,57,58]. Although vitamin D deficiency is common with NAFLD and NASH, data for the effectiveness of vitamin D supplementation has been unclear [53,56,58]. Nobili and Reif suggested that vitamin D may induce anti-fibrotic effects by suppressing hepatic stellate cell proliferation [54]. Sharifi et al. noted that vitamin D therapy reduced inflammatory markers in NAFLD such as C-reactive protein and malondialdehyde [59]. Despite this, multiple studies have failed to find a beneficial response to vitamin D supplementation in liver function or histology in patients with NAFLD [53,56,58]. Furthermore, vitamin D therapy is clinically limited due to its effect on calcium homeostasis and potential for hypercalcemia, a risk factor for NAFLD [53,60]. Vitamin A has not been studied extensively in patients with NAFLD, but like Vitamin D, a significant number of patients with NAFLD have been noted to have vitamin A deficiency [61,62].

### 5.2. Minerals

**Figure 2:**
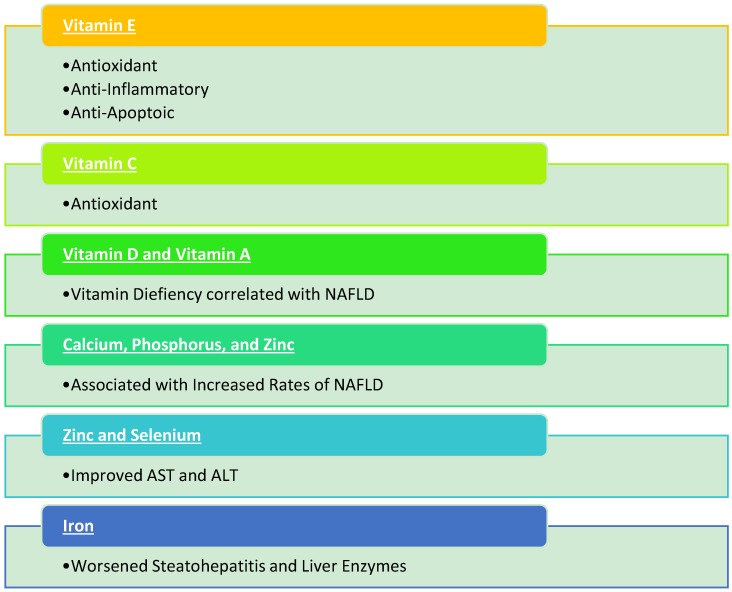
Summary of vitamins and minerals with nonalcoholic fatty liver disease (NAFLD).

Commonly grouped with vitamins, minerals may provide a therapeutic benefit in patients with NAFLD and/or NASH. However, studies are lacking. Cross-sectional analyses have noted that calcium, phosphorus, and sodium intake have been associated with increased rates of NAFLD while magnesium has been inversely related with NAFLD [60,63,64]. In animal studies, reduction in AST and ALT in mice with NAFLD was noted following zinc and selenium co-supplementation [65,66]. Clinical studies are needed to assess whether supplementation would be beneficial for NAFLD patients. Another animal study noted that iron supplementation worsens steatohepatitis with increased liver enzymes, steatosis, and hepatic inflammation [67]. Figure 2 summarizes the vitamins and minerals discussed in this article.

### 5.3. Other Emerging Treatment Strategies 

In addition to vitamins and minerals, pharmacological therapeutic options have been tested and multiple are currently in phase 3 trial review [68,69]. Selonsertib (GS-4997), a selective inhibitor of apoptosis signal-regulating kinase 1 (ASK1), has been associated with anti-inflammatory and anti-fibrotic effects and is being assessed in phase 3 trials for its efficacy in patients with NASH with bridging fibrosis or cirrhosis (STELLAR3; NCT03053050), (STELLAR4; NCT0305306) [68,69]. Cenicriviroc (CVC) is an antagonist of the C-C motif chemokine receptor 2/5 (CCR2 and CCR5) and has been associated with anti-inflammatory and anti-fibrotic effects, as well as improved insulin sensitivity. It is currently being assessed for the treatment of NASH with stage 2 or 3 fibrosis (AURORA; NCT03028740) [68,69].

## 6. Conclusions

In conclusion, the antioxidant, anti-inflammatory, and anti-apoptotic properties of vitamin E accompanied by its favorable clinical profile have made vitamin E a pragmatic therapeutic choice in non-diabetic patients with histologic evidence of NASH if diet and lifestyle modifications fail to yield any benefits. Efforts to study the utility of vitamin E in combination with other anti-fibrotic agents that may yield additive or synergistic therapeutic effects should continue. 

## Figures and Tables

**Table 1:** Major clinical studies of vitamin E therapy for NAFLD/NASH.

Reference	N	Therapy	Vitamin E Dose Daily	Compared to Control/Placebo	
				Biochemical Improvement	Histological Improvement
Lavine [21]	11	Vit E	400–1200 IU	AST, ALT	N/A
Kawanaka [22]	10	Vit E	300 mg	AST	N/A
Vajro [23]	14	Low Calorie Diet + Vit E	400 mg, 300 mg	ALT	N/A
Ersoz [24]	28	Vit E + Vit C	600 IU	ALT	N/A
Nobili [25]	90	Diet + Exercise + Vit E	600 IU	ALT	N/A
Wang [26]	19	Vit E	100 mg	ALT	N/A
Zohrer [28]	40	Lifestyle + DHA + CHO + Vit E	N/A	ALT	N/A
Hasegawa [15]	20	Vit E	300 mg	ALT	St, LI, Fi
Bugianesi [31]	28	Vit E	800 IU	NS	NS
Dufour [32]	15	Ursodeoxycholic Acid + Vit E	400 IU	ALT	St
Yakaryilmaz [33]	9	Vit E	800 mg	AST, ALT	St
Nobili [34]	25	Vit E and Vit C	600 IU	AST, ALT	St, LI, HB
Sanyal [35]	84	(PIVENS Trial) Vit E	800 IU	AST, ALT	St, LI
Lavine [37]	58	(TONIC Trial) Vit E	800 IU	ALT	HB
Pietu [38]	101	Ursodeoxycholic Acid + Vit E	500 IU	AST, ALT	St, LI, Fi, HB
Kawanaka [40]	23	Vit E + Vit C	300 mg	ALT	LI, Fi
Murer [41]	23	Vit E + Vit C + Selenium	400 IU	ALT	NS

Vit E = Vitamin E; DHA = Docosahexanoic Acid; CHO = Choline; Vit C = Vitamin C; NS = Not significant; N/A = Not applicable; St = Steatosis; LI = Lobular Inflammation; Fi = Fibrosis; HB = Hepatocyte Ballooning.
